# Metformin attenuates post‐epidural fibrosis by inhibiting the TGF‐β1/Smad3 and HMGB1/TLR4 signaling pathways

**DOI:** 10.1111/jcmm.16398

**Published:** 2021-02-21

**Authors:** Zeyuan Song, Tao Wu, Jinpeng Sun, Haoran Wang, Feng Hua, Yap San Min Nicolas, Rupesh KC, Kun Chen, Zhen Jin, Jun Liu, Mingshun Zhang

**Affiliations:** ^1^ Department of Orthopaedics The Second Affiliated Hospital of Nanjing Medical University Nanjing China; ^2^ Department of Emergence Medicine Sir Run Run Hospital Nanjing Medical University Nanjing China; ^3^ NHC Key Laboratory of Antibody Technique Department of Immunology Nanjing Medical University Nanjing China

**Keywords:** epidural fibrosis, HMGB1, metabolomics, metformin, spine operation, TGF‐β1

## Abstract

Excessive post‐epidural fibrosis is a common cause of recurrent back pain after spinal surgery. Though various treatment methods have been conducted, the safe and effective drug for alleviating post‐epidural fibrosis remains largely unknown. Metformin, a medicine used in the treatment of type 2 diabetes, has been noted to relieve fibrosis in various organs. In the present study, we aimed to explore the roles and mechanisms of metformin in scar formation in a mouse model of laminectomy. Post‐epidural fibrosis developed in a mouse model of laminectomy by spinous process and the T12‐L2 vertebral plate with a rongeur. With the administration of metformin, post‐epidural fibrosis was reduced, accompanied with decreased collagen and fibronectin in the scar tissues. Mechanistically, metformin decreased fibronectin and collagen deposition in fibroblast cells, and this effect was dependent on the HMGB1/TLR4 and TGF‐β1/Smad3 signalling pathways. In addition, metformin influenced the metabolomics of the fibroblast cells. Taken together, our study suggests that metformin may be a potential option to mitigate epidural fibrosis after laminectomy.

## 1 INTRODUCTION

With the ageing of the population and the increase in sedentary work, low back pain has gradually become a health problem that threats millions of people. Laminectomy is one of the most common surgical methods used after the failure of conservative treatment in patients with spinal degenerative diseases.^1^ Approximately, 8% to 48% of patients who undergo lumbar disc surgery develop failed back surgery syndrome (FBSS).^2^, ^3^ Excessive epidural scar adhesion is a common cause of FBSS. Scars can compress or stretch the nerve roots or dura mater, which may lead to a series of corresponding symptoms, such as persistent low back pain.^4^

Epidural scar adhesion is a common complication after laminectomy. Scar formation consists of three stages: inflammation, granulation tissue formation and scar remodelling. In the inflammatory reaction stage, a large number of inflammatory cells are recruited to the wound or lesion. Various cytokines secreted by these inflammatory cells boost the differentiation of fibroblasts into myofibroblasts. It can also promote the proliferation of local fibroblasts and the deposition of a large amount of extracellular matrix (ECM) components, such as collagen and fibronectin.^5^, ^6^ With the excessive accumulation of collagen, granulation tissue is gradually formed, which eventually leads to the formation of epidural scars. There are many drugs currently used to prevent the formation of epidural fibrosis, such as mitomycin C,^7^ non‐steroidal anti‐inflammatory drugs^8^ and immunosuppressive agents.^9^ Though some types of medicines above have shown the effectiveness among animal experiments, the toxicity of these drugs hinders their clinical application.^10^, ^11^

Metformin is a biguanide oral hypoglycemic agent and is widely used among patients with type 2 diabetes. Sufficient evidence has shown that metformin also has strong anti‐inflammatory, antioxidant and antitumor functions.^12^, ^13^, ^14^ Furthermore, metformin can reduce fibrosis in many organs. For example, metformin reverses established lung fibrosis by promoting myofibroblast inactivation and apoptosis.^15^ Metformin is also capable of reducing liver and renal fibrosis.^16^, ^17^ The role for TGF‐β1 in the development of multi‐organ fibrosis has been widely accepted.^6^ TGF‐β1 is a pluripotent growth factor that plays an important role in regulating the generation of ECM and the development of organ tissue fibrosis. The downstream trans‐membrane TGF‐β1 receptors are multiple Smad proteins, and Smad3 is one of the important signalling molecules that contribute to fibrosis. As an effective inhibitor of TGF‐β1 signalling,^18^ metformin can reduce cardiac fibrosis by inhibiting TGF‐β1/Smad3 signalling.^19^

High mobility group box 1 (HMGB1) is a multi‐functional protein involved in inflammatory reactions and is known to play a key role in tissue repair and fibrosis.^5^ It has been reported that HMGB1 bound to Toll‐like receptor 4 (TLR4) promotes fibrosis.^20^ Moreover, the blockade of advanced glycation end products or TLR4 inhibited the fibroblast activation induced by HMGB1.^21^ Under surgical stimulation, HMGB1 is secreted and released into the extracellular environment. After binding to specific receptors, it stimulates the production of a large number of inflammatory mediators and participates in inflammation,^22^ which contributes to the early stage of epidural fibrosis.

Though it is known that metformin can reduce organ fibrosis, its corresponding function in post‐epidural fibrosis requires further investigation. Organ fibrosis often involves extensive inflammation. For example, viral myocarditis can cause cardiac fibrosis,^23^ and hepatitis C virus (HCV) can result in liver fibrosis.^24^ Thus, these fibrosis models often require pathogen induction. However, epidural fibrosis results from aseptic inflammation. Therefore, we constructed a murine laminectomy model to observe the effect of metformin on epidural scars. The mechanisms behind the therapeutic effects of metformin on post‐epidural fibrosis, especially regarding the TGF‐β1/Smad3 and HMGB1/TLR4 signalling pathways, were also explored.

## 2 MATERIALS AND METHODS

### 2.1 Patients and clinical samples

Eight patients participated in the study, who underwent laminectomy in the Second Affiliated Hospital of Nanjing Medical University between April 2019 and September 2019. The present study was approved by institutional research ethics committee of the Second Affiliated Hospital of Nanjing Medical University (2019 KY 056) and written informed consent was obtained from each subject. Post‐operative peripheral blood was collected from superficial veins of the upper limb, and the post‐operative drainage fluid was extracted from the drainage tube. Plasma samples were harvested by centrifugation at 400 g for 5 minutes, and the supernatant was stored at −80°C for further analysis.

### 2.2 Cell culture

The NIH‐3T3 cell line was obtained from the American Type Culture Collection (ATCC, CRL‐1658) and cultured in DMEM (HyClone, GE) with 10% foetal bovine serum (Lonsera) and 1% penicillin/streptomycin (HyClone, GE) at 37°C in 5% CO_2_ according to the protocol provided by the ATCC. The NIH‐3T3 cells were cultivated in 24‐well plates (1 × 10^5^ cells/well). The next day, the cells were stimulated with the following agents: 10 mmol/L metformin (D150959‐5G; Sigma), 250 ng/mL HMGB1 (50913‐M01H; Sino Biological), 10 ng/mL TGF‐β1 (100‐21‐10UG; PeproTech) and 1 ng/mL SRI‐011381 hydrochloride (Smad3 agonist, HY‐100347A; MCE), respectively. In the control group, the cells were treated with the equal volume of PBS (HyClone, GE). The cells were harvested after 48 hours for further analysis.

### 2.3 Western blotting

Total protein from the cells was extracted via cell lysis in RIPA buffer (P0013B; Beyotime) containing 1 mmol/L PMSF (ST506; Beyotime) followed by sonication on ice for 10 seconds. The experiments were repeated independently at least three times. The concentration was determined by a BCA assay. Equal amounts of protein were separated by 10% SDS‐PAGE and transferred onto polyvinylidene difluoride membranes (Millipore). Then, the membranes were blocked in 5% skim milk for 1 hour at room temperature and then incubated at 4°C overnight with the following primary antibodies: anti‐fibronectin (15613‐1‐AP; Proteintech), anti‐TGF‐β1 (ab170874; Abcam), anti‐Smad3 (9513; Cell Signalling Technology), anti‐phospho‐Smad3 (9520; Cell Signalling Technology), anti‐HMGB1 (10829‐1‐AP; Proteintech), anti‐TLR4 (sc‐293072; Santa Cruz) and anti‐β‐actin (4970L; Cell Signalling Technology). The next day, the membranes were washed with TBST and incubated with horseradish peroxidase (HRP)‐conjugated goat anti‐rabbit IgG (EarthOx Life Sciences) or goat anti‐mouse IgG (H + L) HRP (s0002; Affinity Biosciences) for 1 hour at room temperature. Subsequently, the membranes were immersed in Immobilon Western Chemiluminescent HRP Substrate (Millipore), and images were collected by the G:BOX gel doc system (Syngene).

### 2.4 Animal experiments

Male wild‐type C57BL/6J mice aged 6 to 8 weeks were obtained from the Laboratory Animal Center, Yangzhou University and were housed under specific pathogen‐free conditions at Nanjing Medical University. All animal experiments were reviewed and approved by the Institutional Animal Care and Use Committee (IACUC) of Nanjing Medical University.

The mice were anesthetized by the intraperitoneal injection of a mixture of 10 mg/kg xylazine and 200 mg/kg ketamine hydrochloride in 100 μL normal saline. During the laminectomy, the T12‐L2 vertebral plate was exposed with a midline skin incision, and the dura mater was exposed after removing the spinous process and the T12‐L2 vertebral plate with a rongeur. The incisions were sutured by the spinal fascia, muscle and skin. Metformin (1.5 mg/mL) was supplied via the drinking water after the surgery.

### 2.5 Haematoxylin and eosin staining

After 30 days, the wound tissues were harvested and then fixed in 10% buffered formaldehyde solution for 48 hours. Then, the product was decalcified in 10% neutral EDTA for approximately 4 weeks and finally embedded in paraffin. The paraffin blocks were sectioned into 5‐µm‐thick slices by a Leica model 2165 rotary microtome (Leica) and placed on glass slides. The deparaffinized tissues were stained with haematoxylin for 15 minutes, and then washed with distilled water. Afterwards, the tissues were immersed in 1% hydrochloric acid‐alcohol for 5 minutes and stained with eosin for another 5 minutes. After dehydration, the slices were sealed with neutral gum and observed by microscopy.

### 2.6 Masson staining

Masson staining is often used to evaluate changes in fibrosis in epidural fibrosis models.^25^ Collagen staining was performed using a Masson staining kit according to the protocol recommended by the manufacturer (G1006; Servicebio). After deparaffinization, tissue sections were placed in potassium dichromate overnight. After rinsing with distilled water, the sections were placed into a mixture of ferric haematoxylin solution A and solution B at an equal ratio for 3 minutes. Then, they were dipped in liprune acid magenta for 5 minutes after differentiation. The sections were impregnated with molybdophosphate solution for 2 minutes and aniline blue dye for 5 minutes, and then differentiated with 1% glacial acetic acid and dehydrated with anhydrous ethanol. Finally, images were captured by an Olympus IX73 fluorescence microscope using the appropriate lenses and filters (Nikon).

### 2.7 Immunohistochemical staining

The wound tissues were cut into 5‐μm sections and deparaffinized. Slides underwent antigen retrieval with citrate buffer (pH 6.0) in a pressure cooker for 15 minutes followed by cooling on ice for 20 minutes. The endogenous peroxidase activity was inhibited by using a 3% H_2_O_2_ solution for 15 minutes followed by blocking for 30 minutes using 3% BSA in PBS at room temperature. The slides were then incubated at 4°C overnight with specific primary antibodies against TGF‐β1 (ab170874; Abcam) and HMGB1 (10829‐1‐AP; Proteintech). The slides were then incubated with horseradish peroxidase (HRP)‐conjugated goat anti‐rabbit IgG (EarthOx Life Sciences) for 1 hour at room temperature. Then, freshly prepared DAB Chromogenic Reagent to the stained tissues was added. Images at different sections of tissue from each group were acquired at ×400 magnification with a microscope (model BX‐53; Olympus Optical).

### 2.8 Confocal microscopy and immunofluorescence

NIH‐3T3 cells (1 × 10^5^ in a confocal dish) or frozen wound tissue sections were fixed with 4% paraformaldehyde for 30 minutes, washed with PBS containing 0.1% Triton X‐100 for 10 minutes, blocked with 5% goat serum for 1 hour, and incubated with primary antibodies against fibronectin and HMGB1 overnight at 4°C. The next day, the cells or tissues were incubated with the corresponding secondary antibodies in darkness for 2 hours and were counterstained with DAPI (36308ES20; Yeasen). The immunofluorescence was observed with a ZEISS LSM710 confocal fluorescence microscope or an Olympus IX73 fluorescence microscope.

### 2.9 Enzyme‐linked immunosorbent assay

To assess the total collagen level, 40 mg of the wound tissue was homogenized in 400 µL PBS and centrifuged at 12 000 g for 10 minutes at 4°C. The supernatant was harvested and subjected to the collagen I assay kit according to the manufacturer's instructions (E‐EL‐M0325c; Elabscience). The HMGB1 Enzyme‐Linked Immunosorbent Assay kit (ST51011; IBL International) was used to analyse the HMGB1 content in the human peripheral blood and drainage samples. The TGF‐β1 content in the human peripheral blood and drainage samples was measured using the TGF‐β1 Direct Enzyme‐Linked Immunosorbent Assay kit (DB100B; R&D Systems) according to the manufacturer's instructions. The absorbance was measured at 450 and 630 nm, and the concentrations were calculated according to a standard curve.

### 2.10 Magnetic resonance imaging

MRI has a very high resolution and allows the visualization of soft tissue without causing damage to the body. Therefore, MRI can effectively evaluate the degree of adhesion in an epidural scar.^26^ MRI was performed 30 days after the laminectomy. The mice were anesthetized with isoflurane and scanned. The MRI was performed using a Bruker 7.0T Micro‐MR imaging system. For T2‐weighted sagittal spin echo imaging, the repeat time was 3000 ms; the recovery time was 45 ms; the thickness was 0.5 mm; the matrix was 256 × 256, the field of view was 50 mm; and the total time per set was 6 minutes 24 seconds. For T2‐weighted axial spin echo imaging, the repeat time was 4262.5 ms; the recovery time was 45 ms; the thickness was 0.5 mm; the matrix was 256 × 256; the field of view was 35 mm; and the total time per set was 6 minutes 49 seconds 198 ms.

### 2.11 Metabolomics analysis

Each sample was thawed on ice, 1 mL pre‐cooled extractant (70% methanol aqueous solution) was added, and the mixture was shaken for 1 minute. The mixture was frozen for 3 minutes in liquid nitrogen and allowed to thaw for 3 minutes before it was shaken for another 2 minutes. The procedures above repeated three times. The mixture was centrifuged again at 12 000 rpm at 4°C for 10 minutes. Finally, the supernatant was placed into the sample bottle for LC‐MS/MS analysis.

The sample extracts were analysed by an LC‐ESI‐MS/MS system (UPLC, Shim‐pack UFLC SHIMADZU CBM A system, https://www.shimadzu.com/; MS, QTRAP^®^ System, https://sciex.com/). The analytical conditions for the UPLC were as follows: column, Waters ACQUITY UPLC HSS T3 C18 (1.8 µm, 2.1 mm × 100 mm); column temperature, 40°C; flow rate, 0.4 mL/min; injection volume, 2 μL; solvent system, water (0.04% acetic acid): acetonitrile (0.04% acetic acid); gradient program, 95:5 V/V at 0 minute, 5:95 V/V at 11.0 minutes, 5:95 V/V at 12.0 minutes, 95:5 V/V at 12.1 minutes, 95:5 V/V at 14.0 minutes.

LIT and triple quadrupole (QQQ) scans were acquired on a triple quadrupole‐linear ion trap mass spectrometer (QTRAP) QTRAP^®^ LC‐MS/MS System equipped with an ESI Turbo Ion‐Spray interface, operating in positive and negative ion mode and controlled by the Analyst 1.6.3 software (SCIEX). The ESI source operation parameters were as follows: the source temperature was 500°C; the ion spray voltage (IS) was 5500 V (positive) and −4500 V (negative); the ion source gas I (GSI), gas II (GSII) and curtain gas (CUR) were set at 55, 60 and 25.0 psi, respectively; and the collision gas (CAD) was high. Instrument tuning and mass calibration were performed with 10 and 100 μmol/L polypropylene glycol solutions in the QQQ and LIT modes, respectively. A specific set of MRM transitions was monitored for each period according to the metabolites eluted within the period.

The data provided in this article have been deposited to the EMBL‐EBI MetaboLights database with the identifier MTBLS2092. The complete dataset can be accessed at the following URL: https://www.ebi.ac.uk/metabolights/MTBLS2092.

### 2.12 Statistical analysis

Statistical analyses were carried out using the GraphPad Prism 8.0 software. The data are expressed as the mean ± SEM. Two‐tailed and unpaired *t* test was used to compare the differences between two groups, whereas one‐way ANOVA was performed to determine the significance among more than two groups. Statistical significance was defined as follows: *, *P* < .05; **, *P* < .01; ***, *P* < .001; and NS, not significant.

## 3 RESULTS

### 3.1 Metformin alleviated epidural fibrosis after laminectomy

**FIGURE 1:**
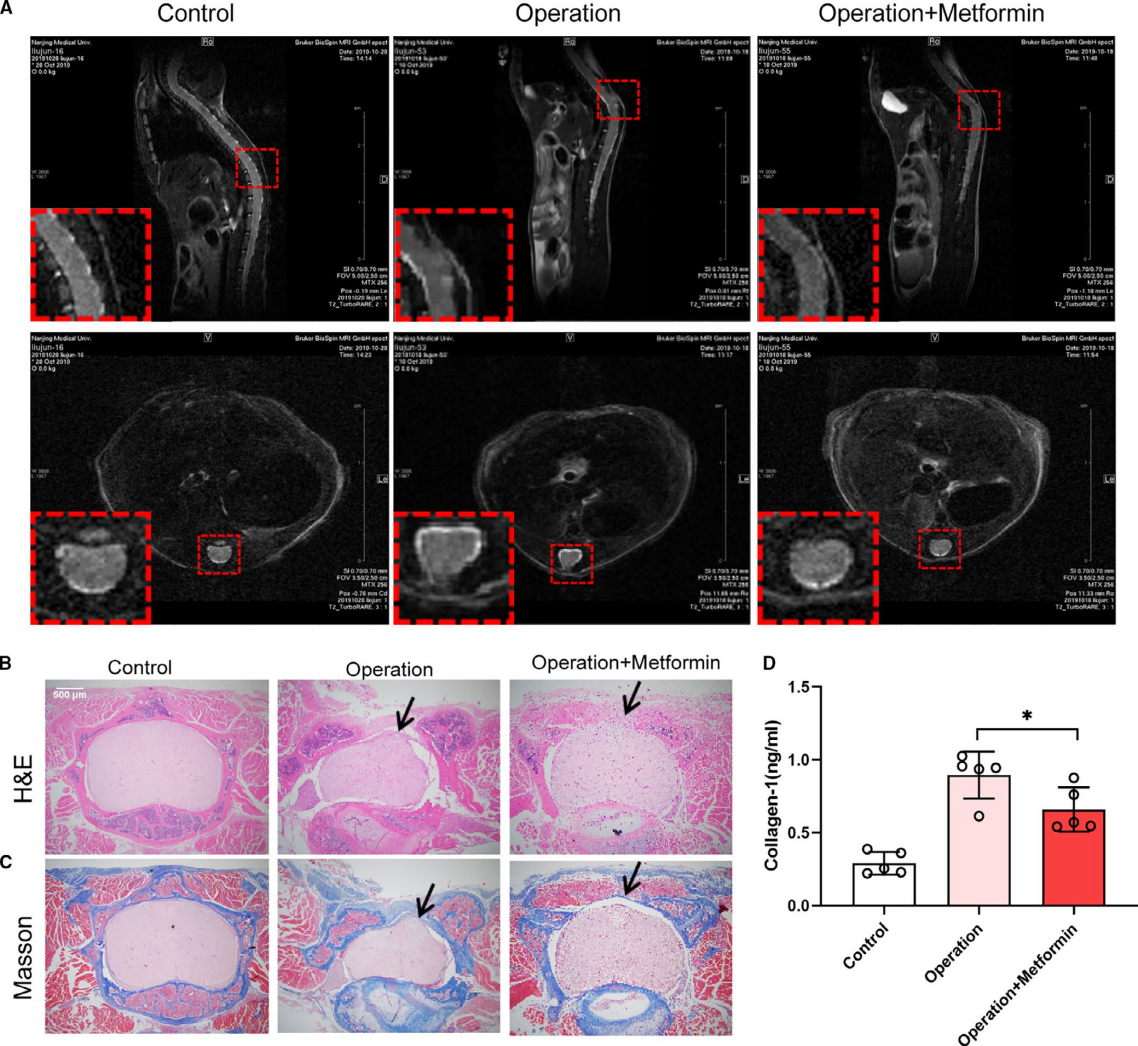
Metformin alleviated epidural fibrosis after laminectomy. (A) MRI observation of epidural fibrosis in mice 30 days after laminectomy. MRI showed that there was a lacuna between the scar and dura mater after the metformin treatment, indicating that the degree of scar adhesion decreased. (B) Image of an epidural scar with H&E staining. The black arrow indicated the scar. (C) Image of an epidural scar with Masson staining. The black arrow indicated the scar. (D) Collagen I assay of wound tissue after laminectomy. Metformin decreased collagen I content in the scar tissue of the operation group. *, *P* < .05

On the 30th day after the laminectomy, MRI analysis showed that severe epidural scar adhesions were produced after laminectomy in the mice and even protruded into the muscle tissue outside the lamina, whereas the dura adhesions were alleviated in the mice in the metformin treatment group (Figure 1A). To obtain an optimal visualization of the laminectomy site, the mice were sacrificed to dislodge intact wound tissue, which was processed for histological analysis after MRI examination. As expected, abundant epidural fibrosis and the excessive proliferation of collagenous tissue were observed between the dura mater and the paraspinal tissue, which adhered to the dura mater, in the non‐treatment group. However, in the mice treated with metformin, there was little or no dural adhesion at the laminectomy site (Figure 1B). The level of proliferation of collagenous tissues in the metformin‐treated mice was significantly lower than the control mice (Figure 1C). Moreover, the collagen I assay showed a significant reduction in collagen content in response to the metformin treatment (Figure 1D). In summary, metformin can effectively relieve scar formation in laminectomy mice.

### 3.2 Metformin decreased fibronectin expression in fibroblasts and epidural fibrosis after laminectomy

**FIGURE 2:**
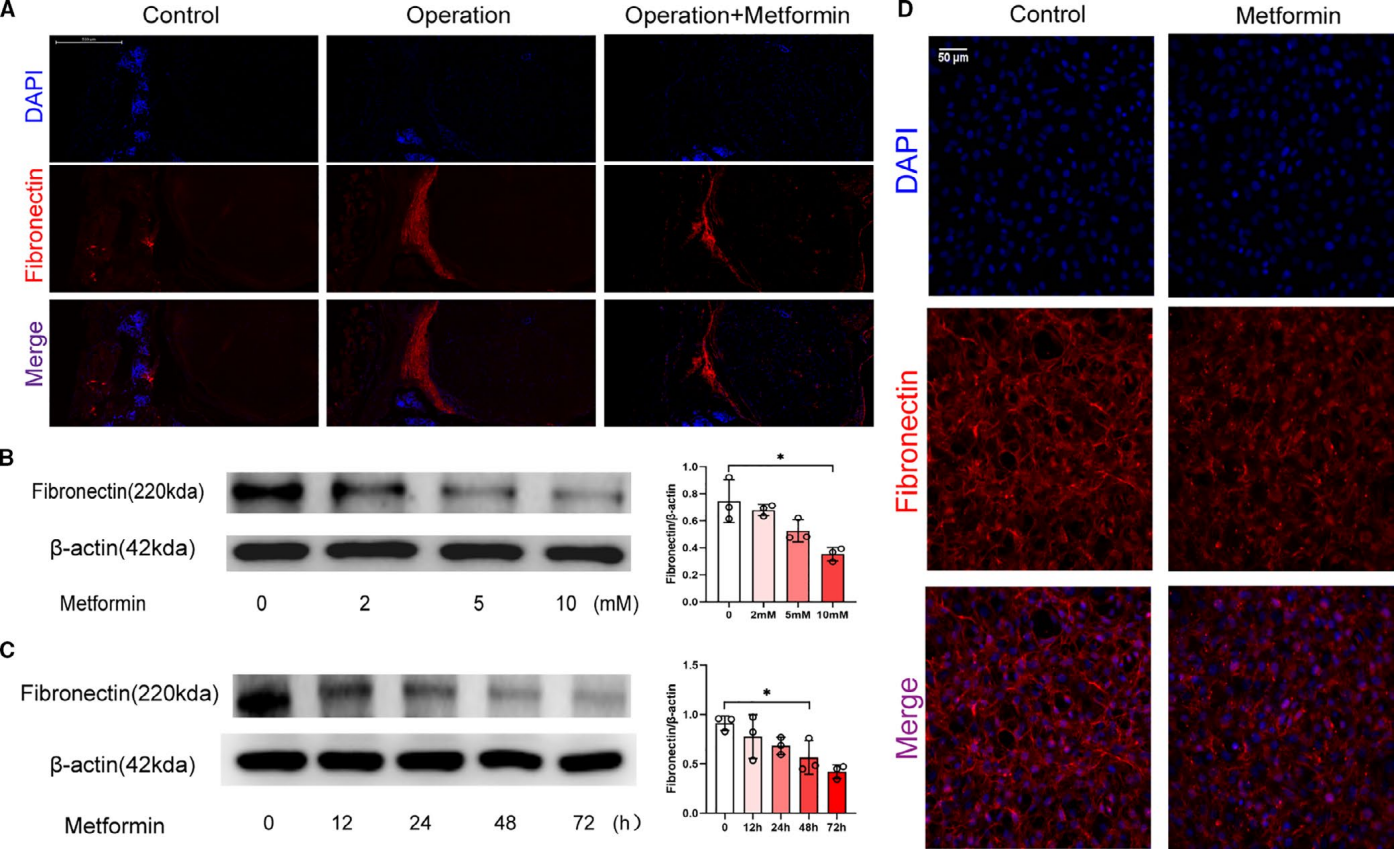
Metformin decreased ECM production in fibroblasts and epidural fibrosis after laminectomy. (A) Frozen sections of the wound tissues were stained with DAPI (pseudo‐blue) and an anti‐fibronectin antibody (red) and observed under a fluorescence microscope. (B) Western blot showing the expression of fibronectin in NIH‐3T3 cells treated with metformin at different concentrations for 48 hours; 10 mmol/L metformin showed a significant reduction. *, *P* < .05. (C) Western blot showing the expression of fibronectin at different time‐points in NIH‐3T3 cells treated with 10 mmol/L metformin. There was a significant difference at 48th hour. *, *P* < .05. (D) NIH‐3T3 cells cultured in 10 mmol/L metformin for 48 hours and stained with DAPI (pseudo‐blue) and an anti‐fibronectin antibody (pseudo‐red) followed by laser confocal microscopy. Metformin destroyed the structure of fibronectin in the NIH‐3T3 cells

As many researchers claimed that fibronectin contributed to post‐epidural fibrosis,^27^ we explored fibronectin expression in the scar tissues in this study. Compared with control group, there was a significant lower expression of fibronectin in scar tissue of metformin‐treated mice (Figure 2A). In order to investigate whether metformin affected fibronectin accumulation, we conducted a dose escalation experiment. NIH‐3T3 cells were treated with 2, 5 or 10 mmol/L metformin for 48 hours. Treatment of cells with 10 mmol/L metformin resulted in the significant down‐regulation of fibronectin expression (Figure 2B). No induction of apoptosis was observed in response to the metformin treatment (10 mmol/L). Therefore, we decided to use the 10 mmol/L concentration for the rest of the study. To further understand the dynamics of this process, we treated NIH‐3T3 cells with 10 mmol/L metformin and analysed samples at 12th, 24th, 48th and 72th hour separately. As observed, fibronectin expression was significantly decreased at the 48th hour (Figure 2C). Thus, we decided to do subsequent analyses for this point. The immunofluorescence staining demonstrated more visually convincing evidence of the substantial decrease in fibronectin expression (Figure 2D). Accordingly, these results suggested that metformin attenuated epidural fibrosis after laminectomy via the reduction in fibronectin.

### 3.3 Metformin reduced TGF‐β1 and HMGB1 expression in vivo and in vitro

**FIGURE 3:**
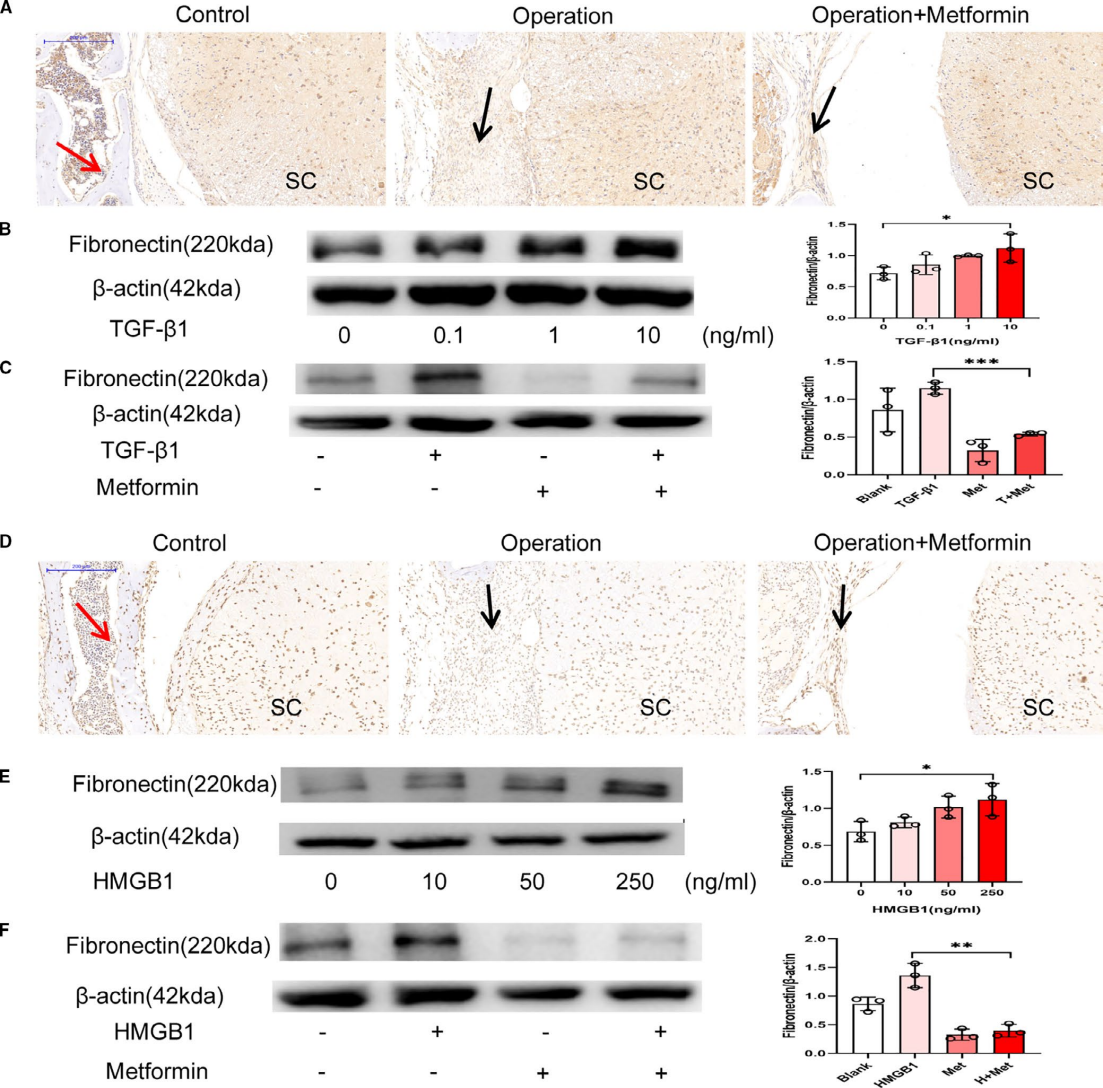
Metformin reduced TGF‐β1 and HMGB1 levels. (A) Immunohistochemical staining images of TGF‐β1 after laminectomy; the red arrow indicates the lamina, the black arrow indicates the epidural scar, and ‘SC’ indicates the spinal cord. The TGF‐β1 content in the metformin group was significantly lower than that in the operation group. (B) Western blot showing the expression of fibronectin in NIH‐3T3 cells treated with TGF‐β1 at different concentrations for 48 hours; treatment with 10 ng/mL TGF‐β1 clearly induced fibronectin expression. *, *P* < .05. (C) Metformin mitigated the effects of TGF‐β1 on the expression of fibronectin in NIH‐3T3 cells. ***, *P* < .001. (D) Immunohistochemical staining images of HMGB1 after laminectomy; the red arrow indicates the lamina, the black arrow indicates the epidural scar and ‘SC’ indicates the spinal cord. Metformin treatment reduced the number of HMGB1‐positive cells. (E) Western blot showing the expression of fibronectin in NIH‐3T3 cells treated with HMGB1 at different concentrations for 48 hours; treatment with 250 ng/mL HMGB1 promoted the expression of fibronectin. *, *P* < .05. (F) Metformin mitigated the effects of HMGB1 on the expression of fibronectin in NIH‐3T3 cells. **, *P* < .01

Furthermore, we explored the causes of fibronectin expression and the mechanisms of metformin in epidural fibrosis. In the immunohistochemical staining analysis, metformin lowered the protein levels of TGF‐β1 in the laminectomy model (Figure 3A), implying that TGF‐β1 may contribute to post‐epidural fibrosis. Indeed, TGF‐β1 boosted fibronectin expression and fibrosis in NIH‐3T3 cells (Figure 3B). In line with the in vivo observations, metformin reduced the fibronectin generation induced by TGF‐β1 (Figure 3C).

Similarly, HMGB1 was detected in the dura and had accumulated in the cytoplasm in epidural scars; however, HMGB1 expression was reduced by the metformin treatment (Figure 3D). Treatment of NIH‐3T3 cells with HMGB1 resulted in the obvious up‐regulation of fibronectin (Figure 3E). As expected, metformin reduced the expression of fibronectin induced by HMGB1 (Figure 3F). In summary, metformin reduced fibronectin via the reduction of TGF‐β1 and HMGB1 in the laminectomy model.

### 3.4 Metformin inhibited TGF‐β1/Smad3 signalling

**FIGURE 4:**
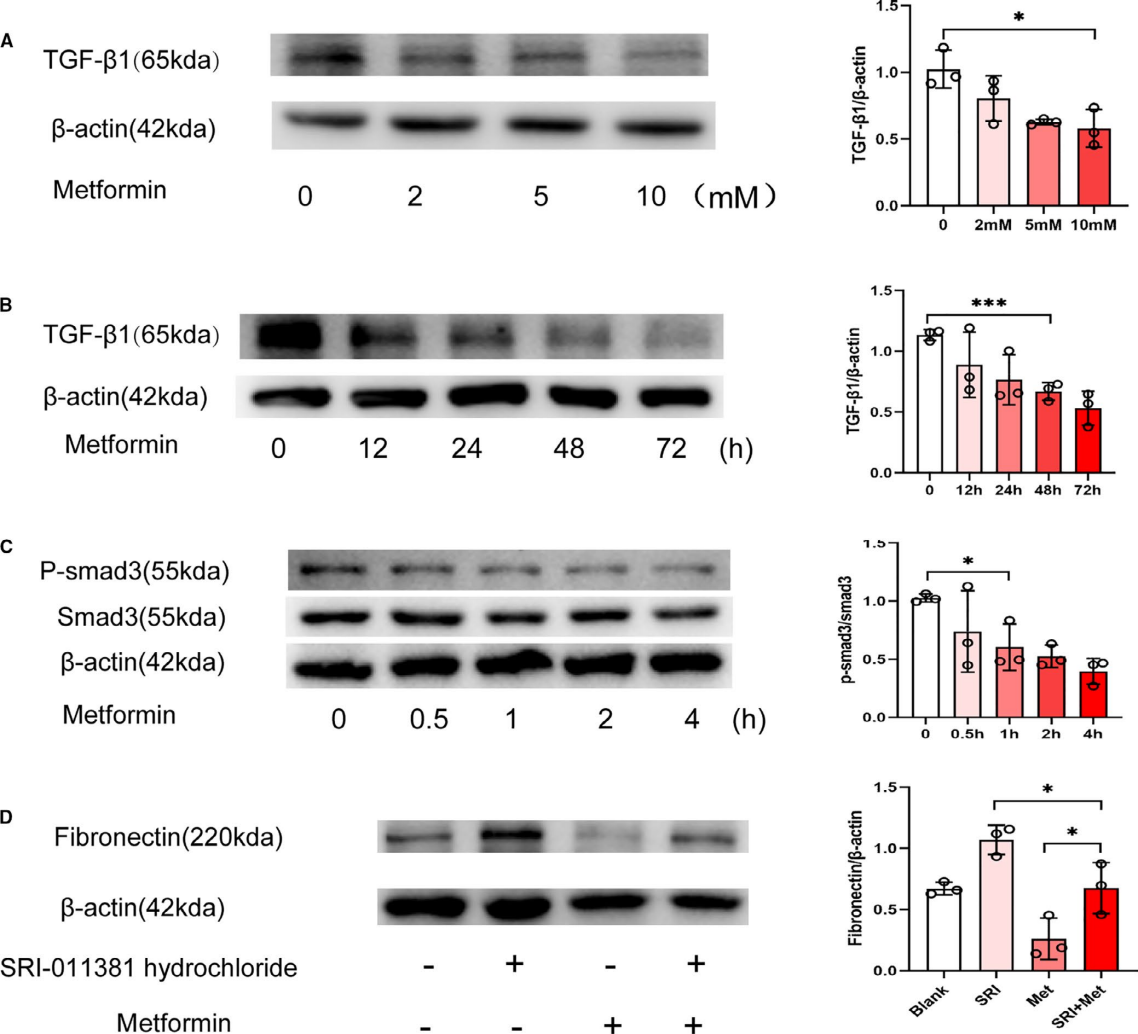
Metformin inhibited TGF‐β1/Smad3 signalling. (A) Western blot showing the expression of TGF‐β1 in NIH‐3T3 cells treated with metformin at different concentrations. Treatment with 10 mmol/L metformin significantly reduced TGF‐β1 levels. *, *P* < .05. (B) Western blot showing the expression of TGF‐β1 at different time‐points in NIH‐3T3 cells treated with metformin. TGF‐β1 was significantly reduced after metformin treatment for 48 hours. **, *P* < .01. (C) Metformin inhibited the phosphorylation of Smad3 in NIH‐3T3 cells. *, *P* < .05. (D) SRI‐011381 (Smad3 agonist) mitigated the effects of metformin on the expression of fibronectin in NIH‐3T3 cells. *, *P* < .05

To discover the specific mechanism by which metformin alleviated post‐epidural fibrosis, we detected TGF‐β1 expression levels by Western blotting. As shown in Figure 4A,B, the expression of TGF‐β1 declined significantly after 48 hours from NIH‐3T3 cells treated by 10 mmol/L metformin. In addition, the phosphorylation of Smad3 in the NIH‐3T3 cells was inhibited by the metformin treatment (Figure 4C). To verify that the metformin‐induced reversal of fibrosis was mediated by TGF‐β1/Smad3 signalling, we treated these cells with metformin in the presence or absence of the Smad3 agonist SRI‐011381 hydrochloride. SRI‐011381 hydrochloride increased fibronectin expression in the NIH‐3T3 cells stimulated by metformin (Figure 4D). These findings suggested that metformin alleviated fibrosis by inhibiting the TGF‐β1/Smad3 signal transduction pathway.

### 3.5 Metformin reduced HMGB1/TLR4 signalling

**FIGURE 5:**
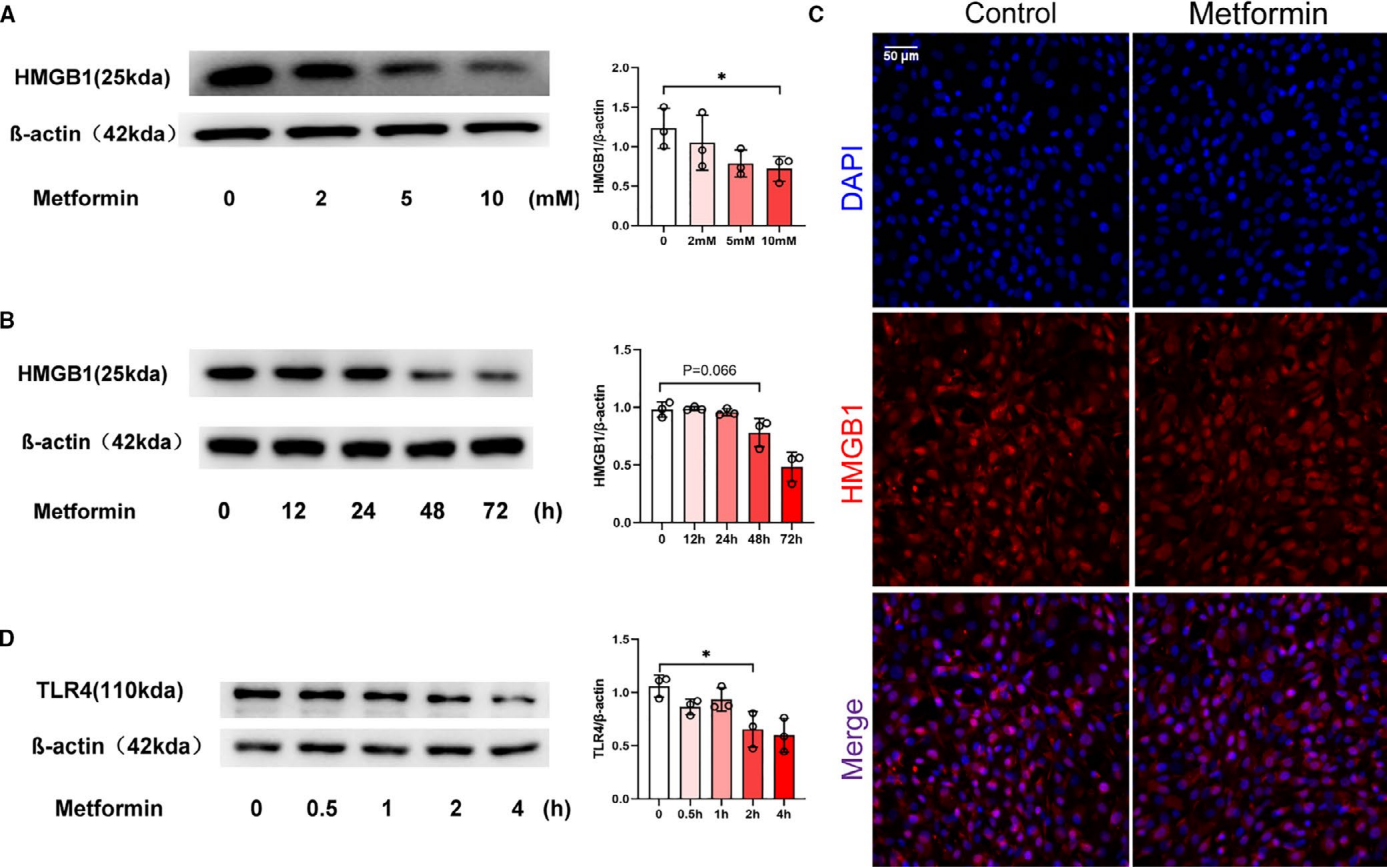
Metformin reduced HMGB1/TLR4 signalling. (A) Western blot showing the expression of HMGB1 in NIH‐3T3 cells treated with metformin at different concentrations. Treatment with 10 mmol/L metformin significantly down‐regulated HMGB1. *, *P* < .05. (B) Western blot showing the expression of HMGB1 at different time‐points in NIH‐3T3 cells treated with metformin. The analysis showed a significant decreased expression of HMGB1 at 48th hour. *P* = .066. (C) NIH‐3T3 cells cultured in metformin for 48th hour and stained with DAPI (pseudo‐blue) and an anti‐HMGB1 antibody (red) followed by laser confocal microscopy. The expression of HMGB1 in NIH‐3T3 cells was reduced after metformin treatment. (D) Western blot showing the expression of TLR4 at different time‐points in NIH‐3T3 cells treated with metformin. The expression of TLR4 began to decrease significantly after metformin treatment for 2 hours. *, *P* < .05

To investigate the effects of metformin on HMGB1 at the cellular level, we cultured NIH‐3T3 cells with metformin. Western blot analysis revealed that treatment with 10 mmol/L metformin for 48 hours decreased the expression of HMGB1 protein in the NIH‐3T3 cells (Figure 5A,B). Fluorescence microscopy showed lower intensity of HMGB1 staining in metformin‐treated cells than those in vehicle‐treated cells after 48 hours (Figure 5C). TLR4, a ligand of HMGB1, was expressed in fibroblasts and closely associated with fibrosis.^28^ As shown in Figure 5D, metformin inhibited TLR4 expression in the NIH‐3T3 cells. In summary, these findings suggested that the metformin‐induced reduction in fibronectin was mediated by HMGB1/TLR4 signalling.

### 3.6 Elevated HMGB1 in clinical samples after laminectomy

**FIGURE 6:**
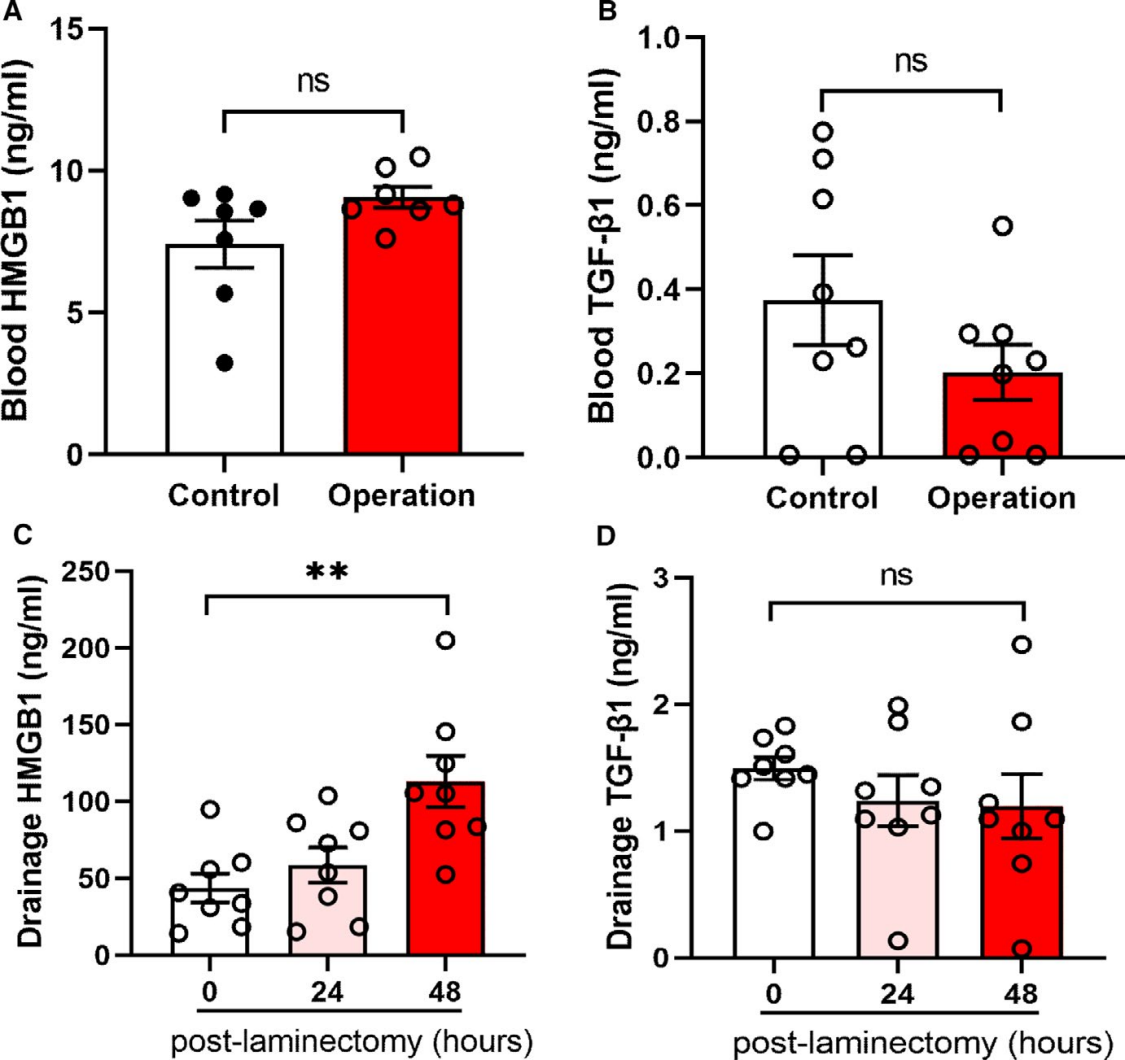
Elevated HMGB1 and TGF‐β1 in clinical samples after laminectomy. (A) HMGB1 content in the peripheral blood of normal and laminectomy patients. NS, not significant. (B) TGF‐β1 content in the peripheral blood of normal and laminectomy patients. NS, not significant. (C) HMGB1 content in the wound drainage fluid from patients undergoing laminectomy. HMGB1 was elevated in the drainage fluid 48 hours post‐laminectomy. **, *P* < .01. (D) TGF‐β1 content in the wound drainage fluid from patients undergoing laminectomy. NS, not significant

To increase the clinical significance of the study, we collected peripheral blood and wound drainage fluid from patients undergoing laminectomy. As shown in Figure 6A,B, the levels of HMGB1 and TGF‐β1 among patients and healthy participants were not significantly different, indicating that the systematic inflammation caused by spinal surgery may be negligible. However, the HMGB1 level improved obviously in the wound drainage after the surgery, reaching the peak concentration 48 hours after operation (Figure 6C). In contrast, the TGF‐β1 level in the early stage after the laminectomy was comparable (Figure 6D). Accordingly, the level of HMGB1 in wound drainage fluid increased after the spinal operation.

### 3.7 Metaformin influenced the metabolism of fibroblast cells

**FIGURE 7:**
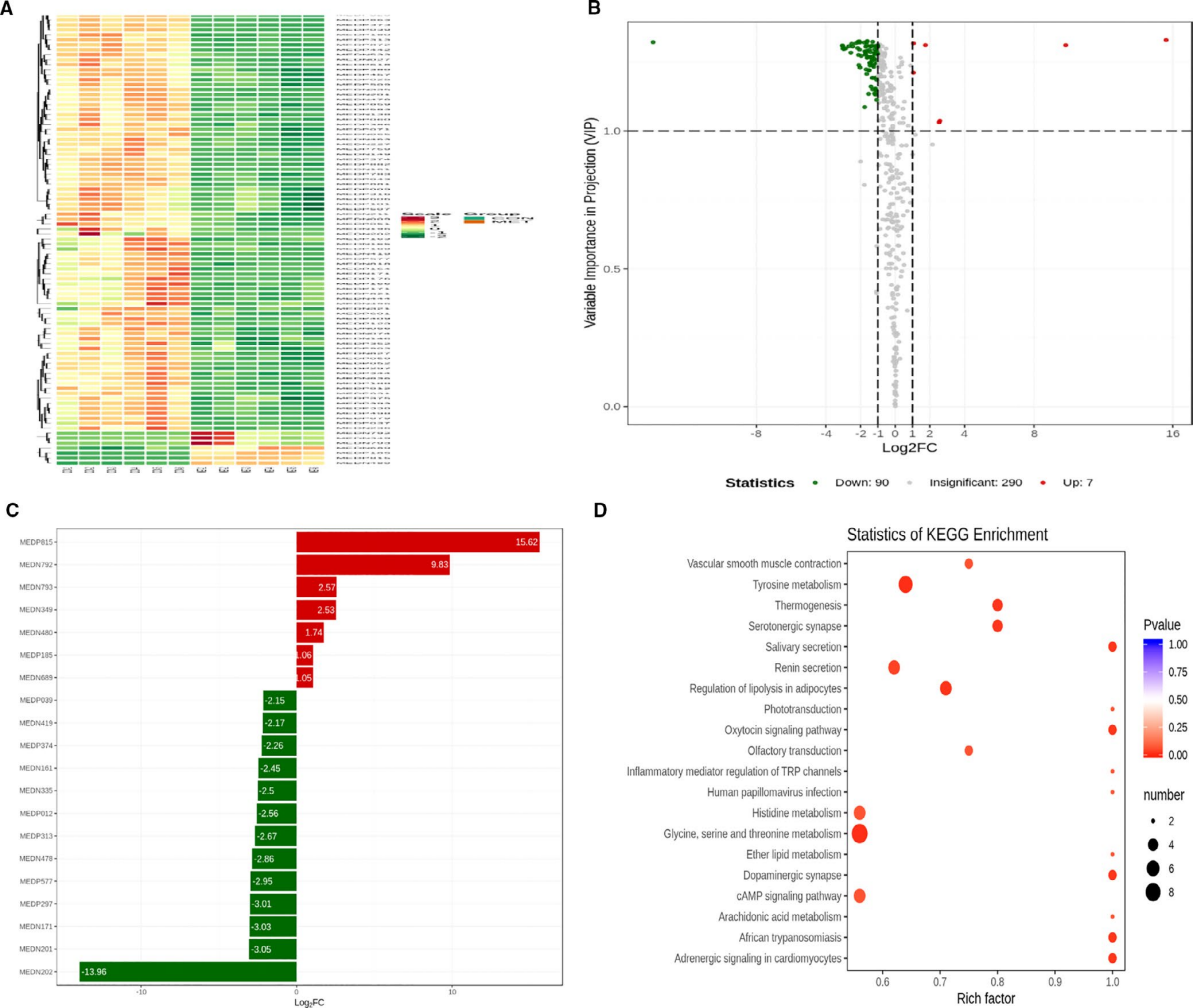
Metformin influenced metabolomics in fibroblast cells. (A) Heatmap presenting the distribution of differential metabolites between metformin‐treated and un‐treated NIH‐3T3 cells. (B) Volcano plots presenting the different levels and statistical significance of metabolites in the supernatants obtained from both groups. (C) Top twenty differential metabolites by the fold‐change value transformed by log2 group between the groups. (D) Overview of the pathway analysis based on the selected supernatant metabolites from the metformin‐treated NIH‐3T3 cells

To investigate the potential roles of metformin in the regulation of metabolism, we analysed metabolomics in NIH‐3T3 cells treated with or without metformin. The integral variations in metabolomics between the normal controls and metformin‐treated NIH‐3T3 cells were assessed in a heatmap (Figure 7A). The differential metabolites in the metformin‐treated NIH‐3T3 group compared with the control group are further presented in a volcano plot analysis (Figure 7B). The top 20 differential metabolites by fold‐change value transformed by log2 were found between the groups (Figure 7C), including 13 down‐regulated metabolites (betaine, uridine 5‐monophosphate, cyclic amp, guanosine 3',5'‐cyclic monophosphate, methylmalonic acid, L‐ornithine, guanidine acetic acid, aminomalonic acid, isobutyryl carnitine, 5‐aminovaleric acid, uridine, succinic acid and a‐ketoglutaric acid). We additionally explored several potential pathways associated with the metformin‐treated NIH‐3T3 cells using pathway enrichment analysis with the Kyoto Encyclopedia of Genes and Genomes database. Tyrosine, glycine, serine and threonine metabolism were significantly enriched in the metformin‐treated NIH‐3T3 cells (Figure 7D). These findings showed that metformin potentially mediated changes in these metabolic pathways.

## 4 DISCUSSION

Epidural scarring refers to scar or tissue fibrosis formed in the wound area after laminectomy, which is the repair response of the body to the wound and one of the most common complications after laminectomy. The inflammatory response and fibroblast proliferation and differentiation play key roles in scar formation. In this study, the mouse laminectomy area produced large amounts of fibronectin and collagen I that resulted in dense epidural scarring. However, the fibronectin and collagen I contents were reduced, and the epidural scar adhesion was relieved after metformin treatment. The data also showed that the treatment of NIH‐3T3 cells with metformin reduced fibronectin expression. These results suggested that metformin inhibited the formation of epidural scarring.

TGF‐β1 promotes fibroblast differentiation into myofibroblasts,^29^ which is an important process of structural modification characteristic of epidural scars. It produces a large amount of ECM components.^6^ Therefore, TGF‐β1 expression is considered to be one of the most important factors in the pathogenesis of epidural fibrosis.^30^ In our experiment, a large amount of TGF‐β1 was detected in the epidural scars after the operation, whereas the amount of TGF‐β1 was significantly reduced after metformin treatment due to the sparse epidural scars. TGF‐β1 expression was significantly decreased in the NIH‐3T3 cells treated with metformin, in line with previous observations that metformin reduced the TGF‐β1‐induced expression of fibronectin.^31^, ^32^ Smad3 is one of the major transcription factors in the TGF‐β1 signalling pathway. In fibroblasts, the regulation of myofibroblast differentiation by TGF‐β1 is mediated by the phosphorylation of Smad3.^19^, ^33^ As expected, metformin lowered p‐Smad3 levels in the NIH‐3T3 cells. In summary, metformin decreased fibronectin expression via the suppression of the TGF‐β1/Smad3 signalling pathway in epidural fibrosis.

Under appropriate stimulation,^34^ HMGB1 is released from the nucleus into the cytoplasm and secreted into the extracellular environment. When extracellular HMGB1 binds to specific receptors of host target cells, it triggers the release of inflammatory mediators and acts an essential part in the inflammatory response.^21^, ^35^ Moreover, HMGB1 can bind to the transmembrane protein TLR4 to induce fibroblast activation and promote fibrosis.^20^, ^21^ Therefore, HMGB1 is involved in the pathogenesis of various organ fibrosis diseases, including myocardial, renal, hepatic and pulmonary fibrosis‐related diseases.^36^, ^37^, ^38^, ^39^ Similarly, we detected a large amount of HMGB1 in the laminectomy area. Metformin treatment significantly decreased HMGB1 levels in vivo and in fibroblast cells, in line with previous studies showing that metformin directly bound to the C‐terminal acidic tail of HMGB1 and inhibited the extracellular activity of HMGB1 to reduce the inflammatory response.^40^ In our experiment, metformin reduced the expression of TLR4, the receptor for HMGB1. In summary, metformin alleviated epidural fibrosis, and this effect may be associated with the TGF‐β1/Smad3 and HMGB1/TLR4 signalling pathways.

Myofibroblast differentiation is accompanied by changes in cellular metabolism. TGF‐β1 can supply glucose‐derived glycine to meet the amino acid requirements associated with enhanced collagen production in response to myofibroblast differentiation.^41^ In the present study, we demonstrated that metformin changed the levels of a wide range of metabolites in fibroblast cells and that these changes may be associated with the reduction in fibronectin and the alleviation of fibrosis. More studies are warranted to explore the mechanisms of metformin and identify novel targets for the treatment of epidural fibrosis.

In conclusion, metformin inhibited the hyperproliferation of epidural scars after laminectomy via the reduction in fibronectin and collagen deposition by inhibiting the HMGB1/TLR4 and TGF‐β1/Smad3 signalling pathways. Based on these results, we suggest that metformin may be a potential therapeutic option to mitigate epidural fibrosis after laminectomy.

## CONFLICT OF INTEREST

The authors report no declarations of interest.

## AUTHOR CONTRIBUTION

**Zeyuan Song:** Data curation (equal); Formal analysis (equal); Investigation (equal); Methodology (equal); Writing‐original draft (equal). **Tao Wu:** Formal analysis (equal); Investigation (equal); Methodology (equal); Validation (equal); Visualization (equal). **Jinpeng Sun:** Investigation (supporting); Methodology (supporting); Validation (supporting). **Haoran Wang:** Investigation (supporting); Validation (supporting). **Feng Hua:** Investigation (supporting); Validation (supporting). **Yap San Min Nicolas:** Investigation (supporting); Validation (supporting). **Rupesh KC:** Methodology (supporting); Validation (supporting). **Kun Chen:** Investigation (supporting); Methodology (supporting). **Zhen Jin:** Investigation (supporting); Methodology (supporting); Validation (supporting). **Jun Liu:** Conceptualization (equal); Funding acquisition (equal); Project administration (equal); Supervision (equal); Writing‐review & editing (equal). **Mingshun Zhang:** Conceptualization (equal); Funding acquisition (equal); Project administration (equal); Supervision (equal); Writing‐review & editing (equal).

## Data Availability

The data that support the findings of this study are available from the corresponding author upon reasonable request.
